# Connexins and Glucose Metabolism in Cancer

**DOI:** 10.3390/ijms231710172

**Published:** 2022-09-05

**Authors:** Jennifer C. Jones, Thomas M. Bodenstine

**Affiliations:** Department of Biochemistry and Molecular Genetics, College of Graduate Studies, Midwestern University, Downers Grove, IL 60515, USA

**Keywords:** connexin, gap junction, metabolism, glucose

## Abstract

Connexins are a family of transmembrane proteins that regulate diverse cellular functions. Originally characterized for their ability to mediate direct intercellular communication through the formation of highly regulated membrane channels, their functions have been extended to the exchange of molecules with the extracellular environment, and the ability to modulate numerous channel-independent effects on processes such as motility and survival. Notably, connexins have been implicated in cancer biology for their context-dependent roles that can both promote or suppress cancer cell function. Moreover, connexins are able to mediate many aspects of cellular metabolism including the intercellular coupling of nutrients and signaling molecules. During cancer progression, changes to substrate utilization occur to support energy production and biomass accumulation. This results in metabolic plasticity that promotes cell survival and proliferation, and can impact therapeutic resistance. Significant progress has been made in our understanding of connexin and cancer biology, however, delineating the roles these multi-faceted proteins play in metabolic adaptation of cancer cells is just beginning. Glucose represents a major carbon substrate for energy production, nucleotide synthesis, carbohydrate modifications and generation of biosynthetic intermediates. While cancer cells often exhibit a dependence on glycolytic metabolism for survival, cellular reprogramming of metabolic pathways is common when blood perfusion is limited in growing tumors. These metabolic changes drive aggressive phenotypes through the acquisition of functional traits. Connections between glucose metabolism and connexin function in cancer cells and the surrounding stroma are now apparent, however much remains to be discovered regarding these relationships. This review discusses the existing evidence in this area and highlights directions for continued investigation.

## 1. Introduction

The development and progression of cancer involves a growing compilation of genetic and epigenetic alterations which affect cellular function and impart qualities that promote self-sufficiency, survival and motility. Influencing these transformations is an ever-changing metabolic milieu that affects cancer cell behavior, and in turn, is influenced by growth of the cancer itself. Transient periods of hypoxia and nutrient deprivation interplay with production of angiogenic factors and alterations to cellular metabolism. As this progression continues, genetic instability drives metabolic adaptations that promote utilization of available substrates in suboptimal growth conditions. Importantly, changes to cellular metabolism are orchestrated by signaling pathways that increase aggressive behaviors and mediate resistance to therapies. Pioneering work in the field has led to profound insight on how metabolic adaptations drive phenotypic qualities that foster the development of metastatic traits [1,2].

The early work of Warburg focused attention on glucose and the ability of cancer cells to maintain robust anaerobic metabolism, even in the presence of available oxygen, a concept that became known as aerobic glycolysis, or the Warburg effect [3]. Keeping glycolysis rates high allows for accumulation of biosynthetic intermediates, promotes nucleotide and fatty acid synthesis, generates NADPH to support glutathione antioxidant activity, and sustains cellular energy levels through a less efficient means of ATP production. Dampening mitochondrial oxidative metabolism decreases excess accumulation of allosteric inhibitors of glycolysis such as citrate, as well as ATP itself, and reduces the potential for mitochondrial free radical production. A volume of evidence supports a dependence on glucose, and a preference for this carbohydrate in cancer cell survival and growth which remains an area of ongoing examination [4,5,6,7,8,9,10,11].

It is established, however, that not all cancer metabolism is centered on glucose, and while mitochondrial dysfunction can occur, these unique cellular organelles within cancer cells are not defective as had been proposed long ago. Indeed, many reports have characterized cancer cells with high oxidative mitochondrial metabolism [12,13,14,15,16,17,18,19]. In vivo, metabolic adaptation is frequently not a choice but rather a requirement for survival, and cancer cells in solid tumors are often not afforded the opulence of high glucose. As a result, upregulated pathways of fatty acid oxidation, amino acid metabolism and autophagic flux are common [20,21,22,23]. Increased utilization of anaplerotic substrates such as glutamine replenish intermediates of the citric acid cycle and degradation of endocytosed components within the microenvironment provide alternative sources of fuel [24].

Despite this adaptability, the availability of glucose during tumor growth undeniably affects metabolism. Of equal magnitude is the influence exerted by its absence. The ability of cells to reprogram, or switch to and from glycolytic metabolism, relates to a phenomenon described in yeast known as the Crabtree effect where high glycolysis rates actively, and reversibly, inhibit components of mitochondrial metabolism [25]. Although the mechanisms of this effect remain unclear, this concept overlaps and partially explains how cancer cells maintain the metabolism described by Warburg, only to later reverse the effect when metabolically necessary [26,27,28]. Conversely, and in line with this, suppression of glycolysis in cancer cell lines increases mitochondrial metabolism [29,30]. Adding to this intricacy, the density of tissue architecture, extracellular matrix and blood pressure affect nutrient and oxygen diffusion differently [31,32,33]. This makes decreased perfusion of substrates a non-linear and highly unpredictable process that necessitates a wide range of cancer cell metabolic plasticity. Targeting cancer cell metabolism therapeutically is therefore a moving and complex objective [34,35].

Throughout these changes, cancer cells are effective at communicating with each other, with stromal cells of the primary tumor tissue, those of secondary sites, and cells encountered during the metastatic cascade [36]. Thus, cells capable of metastasis are not on a journey of self-isolation, but rather one of facilitated interactions that promote a detrimental endpoint for the host. Altering the balance of gene expression, development of functional mutations, and modifying the roles of existing proteins are all mechanisms by which cancer cells achieve their goals. Within this repertoire of instrumentation lies the connexin; a protein involved in many aspects of cellular communication which possesses the ability to suppress oncogenic qualities, but ominously, is often found repurposed within the cancer cell toolkit [37].

What we know about connexins and their functions in cancer has been exceedingly expanded by the field over the last half-century [37]. Mechanistic detail has propelled the roles of connexins from correlation to causation in many cancer cell processes. However, the picture regarding connexins within the ebb and flow of cancer metabolism is only beginning to come into focus. Attention towards this area is increasing with a focus on its relevance [38]. Defining relationships between connexins and the variability of changing metabolic phenotypes will require the study of interconnectivity between numerous metabolites.

## 2. Connexin Biology

Connexins were originally described as the proteins responsible for the formation of cell–cell membrane channels that allowed for direct exchange of small molecules between cells [39,40]. In humans, connexins comprise a family that includes 21 members categorized into five subgroups (α, β, γ, δ, ε) based on sequence homology. Each member is named for the order of discovery within a subgroup constituting their gene symbol (e.g, GJA1) and commonly referred to by the molecular weight in kilodaltons of the expressed protein (e.g., Cx43) [41].

**Figure 1:**
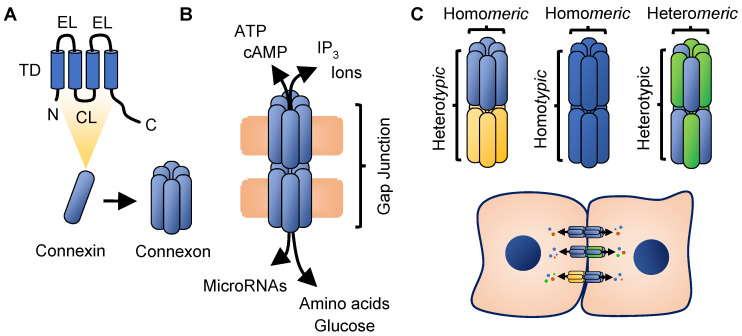
Canonical role of connexins in gap junctional intercellular communication. (**A**) Connexin proteins contain four transmembrane domains (TD), intracellular facing N- and C-terminal regions, two extracellular loops (EL) and one cytoplasmic loop (CL). C-terminal tail and CL are sites of post-translational modifications that affect many aspects of connexin biology. Six connexins form a hexameric channel known as a connexon which forms a highly regulated aqueous pore. (**B**) Connexons are inserted into the plasma membrane and connexons on opposing cell membranes connect to form a gap junction, capable of direct transfer of small molecules and ions. Interactions between ELs are important for formation of gap junctions while other domains mediate different aspects of channel permeability and gating properties. (**C**) Connexons composed of a single type of connexin are termed homomeric while connexons formed from multiple connexins are heteromeric. Gap junctions formed between the same homomeric channels are termed homotypic while connexon docking of differing homomeric channels, or between heteromeric channels are termed heterotypic. The multitude of permutations of these combinations affect the type and directionality of substrate transfer between cells.

Structurally, connexin proteins consists of four hydrophobic transmembrane domains, two extracellular loops, one cytoplasmic loop and intracellular N- and C-terminal domains (Figure 1A). The length of the C-terminal tail represents the greatest divergence among connexins and is a site for numerous protein interactions and post-translational modifications downstream of signal transduction pathways that affect connexin transport and function [42,43,44]. Connexins are co-translated into the endoplasmic reticulum where they are folded and then processed in the Golgi network into hexameric channels known as connexons [45,46,47,48,49]. Connexons are incorporated into the plasma membrane where they connect with connexons on opposing cells to form highly regulated membrane channels that allow for exchange of ions, nutrients and signaling molecules (Figure 1B). This direct cell–cell exchange, known as gap junctional intercellular communication (GJIC), contributes to cellular and tissue homeostasis and is the canonical role described for connexins. Movement from the Golgi to the plasma membrane involves transport along microtubules and interaction with additional cytoskeletal and membrane proteins that facilitate gap junction formation. Interactions with these proteins have led to the discovery of connections with other cellular pathways and functions [50]. Connexins exhibit high turnover rates with half-lives of 1–5 h and degradation involves both the proteasomal and lysosomal pathways [51,52,53,54,55,56,57,58,59,60,61,62,63].

When composed of a single connexin, the resulting connexon channel is said to be homomeric while channels composed of multiple connexins are heteromeric. Compatibility of connexins in this regard is largely dictated by their subgroup categorization (e.g., connexins of the α subgroup pair with other α members) [41,64]. Moreover, gap junctions that form between cells are considered homotypic if composed of identical homomeric connexon units, or heterotypic if the corresponding gap junction is formed from different homomeric connexons, or between any heteromeric connexons (Figure 1C) [65]. Still additional compatibilities exist regarding docking of connexons between cells based on connexin composition, and channel function is responsive to electrical and chemical gating involving the N-terminal, C-terminal, cytoplasmic loop and transmembrane domains, with differences in sensitivity based on the connexin profile [65,66,67]. Collectively, these combinatorial elements affect permeability and directionality of communication, and demonstrate the remarkably high levels of both specificity and activity that can result. Unopposed connexons can also function independently as hemichannels, which mediate communication with the extracellular environment [68,69,70]. Release of molecules such as ATP, NAD^+^ and ions through hemichannels contribute to physiologic processes including purinergic signaling, Ca^2+^ regulation and apoptosis [71,72,73,74,75,76,77,78,79,80]. Expression of connexin genes is found in almost all human cell types with few exceptions, highlighting their necessary roles [81].

The gene structure for most human connexins consists of two exons with all, or most of the coding sequence contained in the second exon [82]. Connexin transcriptional regulation is controlled by basal and tissue specific transcription factors and influenced by second messengers such as cAMP, and hormones including estrogen. Additionally, control of connexin gene expression occurs epigenetically through promotor methylation, histone modifications, and microRNAs (miRNAs), while alternative translation sites generate connexin protein isoforms [83,84,85,86,87,88,89,90,91,92,93,94].

Adding to the complexity of studying connexins, a growing number of roles have been established for channel-independent functions that include effects on cell growth, migration, transcriptional regulation and modulation of signaling pathways (for in depth reviews on these topics see Refs [95,96,97,98,99,100,101,102,103,104,105]). In some cases, effects such as growth suppression can be mediated by only fragments of the connexin protein [106,107]. These expanding functions are due in part to the increasing number of known connexin protein interactions and binding partners (e.g., β-catenin, zonula occludins, Bak, Bcl-XL, cadherins) [42,108]. Thus, the multiplicity of connexin function creates an elaborate background with which to integrate the study of other fields.

## 3. Connexins and Cancer

**Figure 2:**
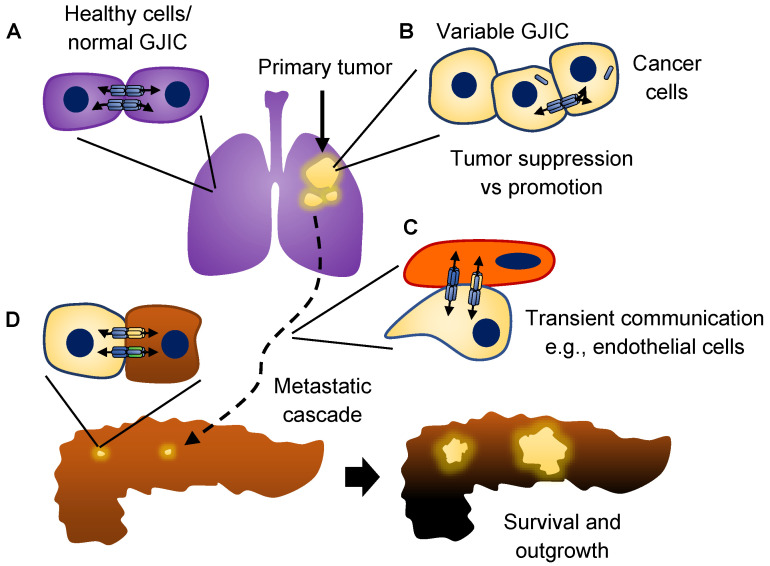
Role of gap junctions during cancer progression. (**A**) GJIC is an essential part of cellular and tissue homeostasis in healthy cells. (**B**) During tumor initiation, loss of GJIC in cancer cells and downregulation of connexin expression is commonly observed. However, the functions of connexins and gap junctions can suppress or facilitate tumor growth. (**C**) During the multi-step process of metastasis, GJIC has been shown to facilitate interactions with other cells (e.g., endothelial cells) to promote the steps of the metastatic cascade. (**D**) Upon reaching a secondary site, adaptation to a new microenvironment must occur and evidence has shown that GJIC between metastatic cells and stromal cells of these sites can promote survival and outgrowth, completing the process of metastasis.

The diverse roles of connexins are firmly intertwined within the complexities of cancer, creating a network of multifarious relationships which have been comprehensively examined in recent excellent reviews to which the reader is referred for a more detailed discussion [37,109,110]. Much work has been accomplished to improve our understanding in this area and continued delineation of these interconnections stands to benefit cancer therapeutics [109,111,112,113,114]. However, accomplishing this goal remains a daunting task. When tumor cells were first reported to have lost electrical coupling through decreased GJIC, a focus emerged on the loss of this communication [115]. The relevance of this association was strengthened by studies which showed direct relationships between the aggressive cancer cell phenotype, cancer stem cell qualities and loss of GJIC [116,117,118,119,120,121,122,123,124,125]. This decrease has been linked to reduced expression of connexin genes, mislocalization of connexin proteins and changes to morphologic and cell–cell adhesive qualities [37]. However, substantial conflicting clinical evidence regarding prognostic implications for expression of connexins in cancer patients served as a prelude to subsequent growing experimental results that proved connexins could play a role in favor of cancer progression and metastasis, in part, through mediating communication with endothelial and stromal cells during the metastatic cascade (Figure 2) [126,127,128,129,130,131,132,133,134,135,136,137,138,139,140,141,142,143,144,145,146,147,148,149]. Explaining these opposing functions is a major effort in the field and has been shown to involve a multitude of context-dependencies. Considering the cause and effect of changes to cancer cell metabolism, a better understanding of how this relates to connexin function and regulation is requisite to continued advancement in this area. The focus of this brief review will be to highlight the current evidence regarding what is known about the connections between glucose metabolism and connexin regulation in cancer cells, emerging evidence, and areas for future directions.

## 4. Connexins and Glucose

### 4.1. Metabolic Coupling of Glucose Metabolism

Due to the ability of gap junctions and hemichannels to allow for transport of small molecules, including glucose, a natural capability to couple metabolism between cells has been established in this regard [150,151,152,153,154,155]. In the study of cancer metabolism, this has many important implications. During periods of glucose availability, glycolysis creates significant lactate production which is exported from cells using monocarboxylate transporter (MCT) proteins that co-transport protons [H^+^], contributing to extracellular acidification of tumor microenvironments. This acidification can eventually dissipate the intracellular/extracellular H^+^ gradient. Furthermore, cells within the core of a solid tumor have limited exposure to the surrounding environment and blood supply, reducing the opportunity for metabolic exchange. This creates a challenge for cancer cells to export metabolites such as lactate and maintain proper intracellular pH.

Work in pancreatic ductal adenocarcinoma cells using three-dimensional growth assays demonstrated that Cx43 gap junction channels facilitate the exchange of lactate directly between cells of growing spheroids by transferring it away from the inner core [156]. This occured due to a hypoxia gradient from the inner portion of the spheroid towards the outermost edges. The movement of lactate between cells through gap junctions allowed for a favorable dispersion to the normoxic spheroid borders. Regarding this later point, lactate can be utilized in aerobic metabolism and this would occur at a greater rate near the more oxygenated tumor border. Transfer of lactate also exerted an alkalinizing effect across the spheroids, increasing intracellular pH to generate a more favorable environment for cell growth. This GJIC buffering effect was expanded to include diffusion of bicarbonate (HCO_3_^−^) from oxygenated peripheral cells to spheroid cores [157]. Taken together, as lactate is transferred away from the core through gap junctions without the co-transport of H^+^, this creates a favorable gradient for HCO_3_^−^ diffusion into the spheroid, coupling and normalizing intracellular pH. Knockdown of Cx43 ameliorated the cell–cell effects in each case. These reports demonstrate a distinctive mechanism of GJIC to promote substrate channeling between cancer cells, alleviating metabolic inhibition and supporting tumor growth. The uniqueness of such a coupling system in the absence of a blood supply, is the fact that gap junctions are the primary mechanism for direct cell–cell exchange of molecules. This underscores the appeal for cancer cells to utilize connexins as a means of molecular networking, contributing to higher-order coordinated function at the multi-cellular level of a tumor. When loss of GJIC is observed in primary tumors, this may therefore relate to differences in vasculature perfusion and changing metabolic pressures.

A study taking a similar approach in colon cancer cells evaluated the roles of GJIC on nutrient facilitation in three-dimensional colon cancer tumor spheroids. Results from this study showed the ability of Cx43 to mediate transfer of glucose from the periphery to the core [158]. This correlated to an increase in ATP production and decrease in the activity of AMP activated protein kinase (AMPK) which is stimulated in response to decreases in nutrient availability and limits biosynthetic pathways. Consequently, this reduced necrosis within the tumor core and demonstrated a means for nutrient facilitation in the absence of vasculature. The results from these studies collectively demonstrate the ability of gap junctions to overcome nutrient limitations and maintain conditions for tumor growth.

Coupling of metabolites also occurs between cancer cells and stromal cells. In a process known as the reverse Warburg effect, cancer cells induce high rates of glycolytic metabolism in surrounding stromal cells, which then produce metabolites such as pyruvate and lactate that are used by the cancer cells, which take on an oxidative metabolism for efficient ATP production [159,160]. This phenomenon is frequently described for cancer associated fibroblasts (CAFs), which are responsive to cancer cell activity within the tumor microenvironment [161]. Therefore, cancer cells cause the stroma to produce usable metabolites for oxidative phosphorylation and this effect can be mediated through gap junctions. Culturing CAFs with A549 or H1299 non-small cell lung cancer (NSCLC) cell lines caused an increase in glycolytic metabolism in the CAFs with a corresponding upregulation of key glycolysis enzymes, glucose uptake and lactate production [162]. This subsequently led to downregulation of E-cadherin and upregulation of N-cadherin in the NSCLC cells, an invasive phenotype consistent with epithelial-to-mesenchymal (EMT) transition. This effect was blocked if CAFs were first incubated with 2-deoxyglucose, an inhibitor of glycolysis. This study also demonstrated that unidirectional GJIC occurred from CAFs to NSCLC cells through Cx43 channels and was responsible for an increase in aerobic metabolites in the NSCLC cells, including pyruvate, acetyl-CoA, citrate and α-ketoglutarate. When GJIC was inhibited with 18α glycyrrhetinic acid or shRNA targeting Cx43, the effects on NSCLC EMT were diminished, while upregulation of GJIC with retinoic acid or Cx43 overexpression led to increased EMT. Thus, NSCLC influenced glycolytic metabolism in CAFs which supported aerobic metabolism in the NSCLC cells directly through GJIC. Cx43 has also been postulated to play a role in supporting metabolic coupling of tumor associated macrophages (TAMs) during the growth of thyroid cancer [163]. In this report, TAMs were coupled to endothelial cells of nearby blood vessels and also with intermingled cancer cells, displaying Cx43 positivity at the plasma membranes. This allowed for propagation of metabolic substrates through TAMs, allowing them to act as nutritional conduits to deeper layers of the stroma and tumor.

Studies such as these demonstrate that cancer cells are capable of coupling not only their own metabolism, but co-opting the metabolism of other cells to fuel their demands through the use of GJIC. This allows for accessibility to glucose and venting of intermediates in the absence of blood perfusion. These processes have significant implications on the establishment of metastatic adaptation when cells extravasate to secondary sites and must adapt to new microenvironments.

### 4.2. Metabolic Rewiring

Among the many protein interactions of the Cx43 C-terminal tail is c-Src (SRC), a non-receptor tyrosine kinase and proto-oncogene which phosphorylates Cx43 and decreases GJIC [164,165,166]. Conversely however, this interaction can also inhibit the activity of c-Src, in part, by binding its inhibitors PTEN and CSK [167,168]. In its oncogenic state, c-Src phosphorylates numerous substrates to promote uncontrolled growth [169,170,171,172]. Included in these targets are enzymes capable of reprograming cellular metabolism to promote glycolysis and decrease oxidative phosphorylation [173,174,175,176,177,178]. Using glioblastoma stem cells (GSCs) and a cell-permeant peptide fragment of Cx43 (TAT-Cx43_266-283_) that reproduces the binding and inhibition of c-Src by full length Cx43, this inhibition was shown to affect GSC qualities [179]. Hexokinase enzymes catalyze the first step of glycolysis by phosphorylating glucose to glucose-6-phosphate. Phosphorylation of hexokinases by c-Src increases enzyme activity. Accordingly, treatment of GSC cells with TAT-Cx43_266-283_ decreased glucose uptake by reducing hexokinase activity. Moreover, this inhibition reduced the ability of GSCs to adapt to different nutrient limitations. When GSCs were grown in media containing glucose and lacking amino acids, cells adapted by increasing expression of glucose metabolizing enzymes hexokinase-II (HK2) and glucose-6-phosphate dehydrogenase (G6PD), while decreased levels of these enzymes, along with GLUT3 (SLC2A3), a glucose transporter highly expressed in the nervous system, were decreased in TAT-Cx43_266-283_ treated cells. The response involving decreased hexokinase-II and GLUT3 was also recapitulated using an orthotopic murine model of glioblastoma. The results indicate potential tumor suppressive roles of Cx43 related to its ability to block adaptation to glucose utilization through inhibition of c-Src. This effect is likely to be context specific between cancers and dependent on c-Src status.

Studies using C6 rat glioma cells have placed an additional focus on hexokinase and gap junction regulation. Hexokinase enzymes can be tethered to the mitochondrial membrane which increases activity, provides a source of ATP and reduces degradation. This mitochondrial association is also a feature of cancer cells, and can contribute to higher glycolytic rates. Increasing GJIC in the C6 cell line with tolbutamide or dbcAMP (which promote GJIC through mechanisms that include increased connexin expression and improved connexin trafficking to the plasma membrane), led to dislocation of both hexokinase I (HK1) and II from the mitochondria to the cytosol [180]. This alteration was associated with a decrease in hexokinase activity and slowed glucose uptake at the stage of phosphorylation, as GLUT1 (SLC2A1) and GLUT3 levels and localization remained unchanged. In another study using the C6 cell line in a xenograft model, expression of Cx30 (GJB6) inhibited expression of GLUT transporters, hexokinase II and pyruvate dehydrogenase kinase 1 (PDK1) [181]. Phosphorylation of the pyruvate dehydrogenase E1 subunit within the pyruvate dehydrogenase complex by PDK1 reduces its ability to oxidatively decarboxylate pyruvate during the formation of acetyl-CoA in the mitochondrial matrix, the committed step of aerobic metabolism of glucose. Decreasing expression of this inhibitory kinase would bypass allosteric regulation and promote greater oxidative phosphorylation while reducing anaerobic flux of pyruvate to lactate. These initial results suggest Cx30 may act to suppress glycolytic activity through yet to be determined transcriptional mechanisms. The effects described above suggest potential channel-independent functions of connexins resulting in metabolic reprogramming and may also be coupled to exchange of metabolites through GJIC, generating a multi-factorial effect on cancer cell metabolism.

### 4.3. Response to Glucose Availability

Important questions exist regarding metabolic substrate switching based on nutrient availability and the function of connexins, gap junctions and hemichannels. A wealth of reports have described the effects of glucose availability and metabolic inhibition on connexin expression and function through experimental models in the context of diabetes and its complications such as diabetic retinopathy and nephropathy. Many of these studies have demonstrated an effect of downregulation on connexin expression and/or gap junction activity in response to high glucose levels [182,183,184,185,186,187,188,189,190,191,192,193,194,195,196,197,198,199,200,201,202,203]. Although the mechanisms leading to these decreases have not been fully characterized, the reported effects encompass multiple connexins (Cx30.2, Cx36, Cx40, Cx43) and have been linked to a wide range of pathways including p38 and RhoA, with effects from miR-1 and the inducible cAMP responsive element modulator (CREM). However, in line with the dynamic and context-dependent nature of connexins, other studies in this area have shown no effect, or an upregulation in response to high glucose concentrations [204,205,206,207,208]. Conversely, other reports have shown the ability of connexins to directly influence aspects of glucose metabolism [155,209,210,211,212,213]. It is therefore clear that connexins are dynamically regulated by availability of glucose and simultaneously contribute to regulation of its metabolism. However, comparatively, much less is known about this variable relationship and the causative effects it has on cancer cells.

In a study bridging these areas, Cx45 (GJC1) was implicated in a potential connection between liver cancer and diabetes [214]. The basis for this study stems from the fact that diabetes is a risk factor for the development of liver cancer and that Cx45 is a marker upregulated in this condition [215,216,217,218]. Cx45 was inducible in multiple liver cancer cell lines in response to an increase in glucose concentration and led to promotion of cell viability and colony formation and a reduction in apoptosis in vitro. Additionally, this led to improved tumor growth when injected subcutaneously in a BALB/c xenograft model. Importantly, the effects induced by high glucose were inhibited with Cx45 knockdown. Additionally, the authors found that Cx45 was upregulated in liver tumor samples when compared to matched adjacent normal tissue. Mechanistically, this upregulation was mediated by the zinc finger transcription factor APA1 (ZNF410) binding to a motif in the Cx45 promoter and was required for the upregulation of Cx45 induced by high-glucose. Moreover, O-GlcNAcylation modification of APA1 was necessary to exert its effects under these conditions.

This later finding is intriguing as O-GlcNAcylation, which involves the posttranslational addition of N-acetylglucosamine (GlcNAc) to affect protein stability and localization, has received much attention for its significant connections to glycolysis and metabolic reprogramming in cancer cells [219,220,221]. In addition to the role of glucose in glycolysis and the pentose phosphate pathway, a portion of available glucose is diverted to the hexosamine biosynthetic pathway (HBP) for carbohydrate modifications of proteins. The divergence point is at the step of fructose-6-phosphate and involves additional input from glutamine and fatty acid metabolism, providing this pathway with nutrient sensing capacities. In cancer, O-GlcNAcylation may be elevated, in part due to increased glycolytic flux when glutamine is available. Enzymes of glycolysis are modified by O-GlcNAcylation, the most notable of which is the tetrameric enzyme phosphofructokinase-1 (PFK1), the rate limiting enzyme of glycolysis. O-GlcNAcylation reduces activity of PFK1, creating greater flux through the upstream pentose phosphate pathway and HBP. The downstream effects on Cx45 following an increase in APA1 O-GlcNAcylation are interesting and place connexin gene regulation within this network. Although outside the scope of a discussion on cancer, it is important to note that some connexins are directly modified by O-GlcNAcylation including Cx40 and Cx43, which may affect interactions between adjacent connexons, thus modifying GJIC dynamics [222,223].

A study from a different perspective evaluated the effects of reduced glucose availability on metastatic breast cancer cells. Using the MDA-MB-231 triple-negative breast cancer cell line, long-term glucose limitations induced the emergence of an adapted population with altered morphology and a significantly increased invasive capacity through Matrigel [224]. These cells matched the proliferative capacity and viability of non-adapted cells grown in higher glucose concentrations. Of note, these cells exhibited improved cell–cell attachment with increased Cx43 protein levels, membrane localization and GJIC. These later findings suggest a role for increased GJIC during invasive responses to nutrient limitations. The upregulation of Cx43 in response to glucose deprivation has also been observed in rat H9c2 cardiomyoblasts [225]. From a basic interpretation, lack of glucose availability may upregulate connexin expression and GJIC in cancer cells as a way to probe for its presence in surrounding cellular networks. Increasing invasiveness could represent a response to move away from sites of substrate limitation in search of more perfused microenvironments while molding subpopulations with improved communicative capacity, metabolic plasticity and invasive potential.

**Figure 3:**
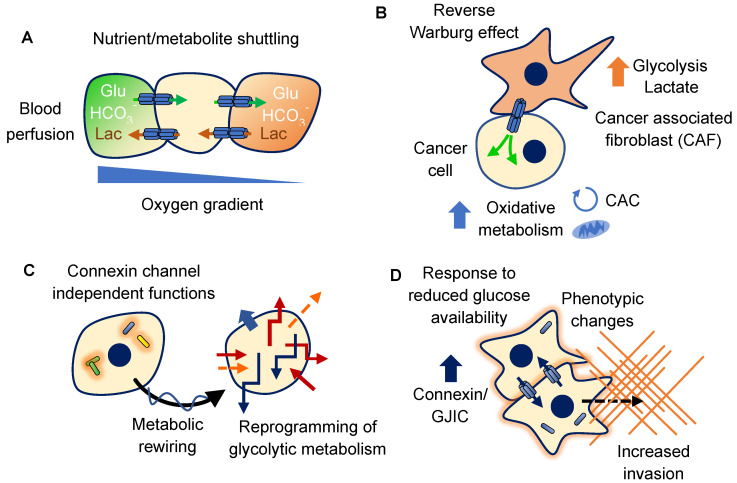
Examples of relationships between glucose availability, metabolism and connexin function. (**A**) Gap junctions allow for exchange of metabolites with a tumor. As tumors grow, regions of hypoxia develop depending on tumor vasculature and angiogenesis. Active GJIC allows for the transfer of glucose and bicarbonate from perfused regions to hypoxic areas. Additionally, metabolites such as lactate can be shuttled to normoxic areas for utilization and reduced buildup, collectively normalizing metabolism in the absence of an integrated blood supply, Refs. [156,157,158]. (**B**) In a process referred to as the reverse Warburg effect, cancer cells induce glycolytic metabolism in stromal cells such as cancer associated fibroblasts (CAFs). This in turn leads to stimulation of oxidative metabolism in cancer cells through the transfer of metabolites. In some models this effect has been shown to be dependent on unidirectional transfer through GJIC, Ref. [162]. (**C**) Connexin expression has been shown capable of controlling metabolic enzymes within cancer cells that affect glycolysis. Although much remains to be determined, many of these effects are mediated through channel-independent functions of connexins. Metabolic reprogramming, or inhibition of this process, affects the adaptability of cancer cells to metabolic changes in the microenvironment, Refs. [179,180,181]. (**D**) Adaptation to reduced glucose availability can lead to increased connexin expression, membrane localization and GJIC with associated increases in phenotypic qualities such as survival and invasion, Refs. [224,225]. Abbreviations: Glu, glucose; HCO_3_^−^, bicarbonate; Lac, lactate; CAC, citric acid cycle. References correspond to related material discussed in this article.

Collectively, these studies demonstrate the ability of GJIC to couple tumor cells metabolically and promote growth within hypoxic regions. They have also shown how connexin functions alter activity of glycoysis and glucose uptake. Importantly, they provide evidence for the promotion of cancer cell qualities in context-dependent responses to high or low glucose, confirming a mutual relationship between the function of connexins and availability of glucose (Figure 3).

## 5. Emerging Connections

### 5.1. Glucose Responsive Degradation Pathways

Changes to the synthesis and processing of connexins in cancer cells may play a role in cancer cell metabolic adaptations. The endoplasmic reticulum associated degradation (ERAD) pathway targets misfolded or damaged proteins within the ER and transports these proteins for ubiquitination and processing by the proteasome. ERAD contributes to turnover of connexins, especially in cells with high expression, or in experimental models involving overexpression [226,227]. Furthermore, processing of multiple connexins through this pathway is mediated by a direct interaction with CIP75 (UBQLN4) [228,229,230]. Interestingly however, the role of ERAD regarding connexins is complex. Upregulation of the ERAD response does not increase ER translocation and degradation of connexins in all cases, while cellular stress can reduce the amount of connexin removed by this pathway leading to increased gap junction formation [227]. The later point may be explained by upregulation of stress-response chaperone proteins that affect ERAD substrate targeting. Cx43 interacts with ERp29 (ERP29), an ER resident protein that facilitates folding and processing of secretory proteins [231]. ERp29 was originally described as a metabolic stress-inducible ER protein and more recent data has demonstrated that ERp29 is downregulated in response to high glucose concentrations by a mechanism involving the glucose-regulated miRNA 483-3p and the long noncoding RNA MEG3 in hepatocellular carcinoma cells [232,233]. This suggests the possibility that ERp29 is induced in response to low glucose which may have subsequent effects on Cx43 transport and degradation. Related to this, ERp29 also affects additional proteins involved in ER degradation including glucose-regulated protein 78 (GRP78; HSPA5), while Cx43 expression has been linked to the potential regulation of additional chaperones including the mitochondrial heat shock protein 60 (HSPD1), and GRP75 (HSPA9), which couples Ca^2+^ transfer between the ER and mitochondria [232,234,235]. Because connexin proteins expressed in cancer cells frequently exhibit transport defects, or are removed from the ER by ERAD, it will be interesting to examine the influence of metabolic shifts on ER processing of connexins.

Glucose availability has clear implications on the process of autophagy, and since connexin proteins are both targets and regulators of this pathway, consideration must be given to how this affects connexin dynamics in cancer cells [236,237,238]. Autophagy promotes cellular survival through breakdown of proteins and cellular organelles for energy utilization under nutrient deplete conditions, in an attempt to sustain cellular activity until availability of usable metabolic substrates is restored. If metabolites remain exhausted, autophagy can induce type II cell death [239]. Thus, autophagy promotes a temporary means of cellular survival and its role in cancer has been widely studied, making it a target for therapeutics [240]. Collectively, continued investigations to delineate these interconnections with connexins and glucose availability will be beneficial to our understanding of potential roles in cancer cell survival, but will also broaden our knowledge on many other pathways that connect, or are affected by, the mechanisms of autophagy.

### 5.2. Mitochondrial Connexins

Cx43 is found within the inner mitochondrial membrane and while much of the functional relevance regarding this localization is unclear, its role has been implicated in complex conditions including cardiac ischemia/reperfusion injury and cell death [241,242,243]. Connections to the influence of glucose metabolism have demonstrated that high glucose downregulates mitochondrial Cx43, leading to changes in mitochondrial morphology, release of cytochrome C and promotion of apoptosis in rat retinal endothelial cells [199,244]. Additionally, transfection of Cx43 increased levels of mitochondrial Cx43 and reduced markers of apoptosis while inhibiting mitochondrial structural changes in response to high glucose, suggesting that mitochondrial Cx43 may play a role in preventing apoptosis due to elevated glucose exposure [244]. Hypoglycemic challenges in cardiomyocytes following long-term hyperglycemia caused loss of Cx43 from the plasma membrane with aberrant accumulation within the mitochondria [245]. The role therefore of mitochondrial Cx43 on the larger scale of cancer, and more specifically, cancer metabolism, has yet to be determined. Given the central connections of mitochondria to aerobic metabolism and cellular substrate utilization, roles for mitochondrial Cx43 in this context are likely to be described.

### 5.3. Integration of Growth Pathways

The phosphatidyl-inositol 3 kinase (PI3K) pathway induces a signaling program that supports cellular growth and survival, and its activity is highly implicated in cancer. Downstream of growth factor binding, PI3K leads to activation of AKT serine/threonine kinases (ATK(1-3)) that phosphorylate numerous targets, collectively promoting cellular survival, energy metabolism and growth [246,247]. Among its substrates are the mammalian target of rapamycin (mTOR) complex I which facilitates cell cycle progression through its inhibitory phosphorylation of 4E-BP1 (EIF4EBP1) and exerts a stimulatory effect on protein translation by promoting activation of S6K1 (RPS6KB1) [248]. PI3K/AKT signaling often occurs in conjunction with other oncogenic growth factor signaling pathways (e.g., RAS/MEK/ERK) which further support a cell growth response and promote the uptake of glucose in a cell-autonomous fashion. Therefore, coupled to the activity of these pathways is substrate utilization. For example, PI3K/AKT leads to direct phosphorylation of multiple glycolysis enzymes to promote glucose utilization, but can also activate alternative pathways in the absence of glucose [249,250,251]. Connexins have been implicated within these signal transduction pathways, including many aspects related to cancer cell function such as motility, invasion, proliferation and therapy resistance [252,253,254,255,256,257,258,259,260]. Ongoing studies drawing connections between these growth promoting pathways and their effects on connexins, will undoubtedly continue to converge on metabolic involvement.

Conversely, connexins are also regulated by nutrient sensing pathways during times of reduced cellular energy availability. AMPK is stimulated when ATP levels decrease and works to inhibit biosynthetic processes while adjusting cellular metabolism based on substrate availability [261]. Connexins have been shown to be targets of AMPK phosphorylation (e.g., Cx26 (GJB2)), indirectly regulate its function, or cooperate with this kinase to bring about cellular changes [262,263,264]. As expected, AMPK activity is highly influenced by glucose which affects its interactions with connexins [265,266]. The challenge of studying growth factor and nutrient sensing pathways such as these however, is determining their individual contributions within a metabolically responsive system.

### 5.4. Hypoxia and HIF1α

Limitations to nutrients such as glucose due to a lack of sufficient blood supply are inherently coupled with hypoxia. Therefore, the study of nutrient limitation effects on cancer cell qualities must be taken into consideration with changing states of oxygen perfusion. HIF1α (HIF1A) is a known response element activated during times of decreased oxygen availability and is highly regulated at the protein level, responding to hypoxia through transcriptional regulation of genes that affect metabolism, and is interconnected with PI3K signaling and cancer [251,267,268,269,270]. HIF1α, along with HIF1β (ARNT) form the heteromeric HIF1 transcription complex. Activity is principally controled at the level of HIF1α which is degraded by the proteasome under normoxic conditions. Decreased oxygen availability reduces breakdown through this pathway and allows HIF1α to heterodimerize with HIF1β and modulate gene expression within the nucleus. Regarding glucose, HIF1 activity increases enzymes for glycolysis as oxidative metabolism of substrates including fatty acids and amino acids will be limited during decreased oxygen availability [271,272]. The first report describing a link between Cx43 and HIF1α involved regulation of glucose metabolism in astrocytes [273]. Cx43 levels were decreased downstream of endothelin-1 (ET-1; EDN1) treatment. This led to an upregulation of HIF1α which was shown to be mediated by c-Src. Since Cx43 is capable of binding and reducing the activity of c-Src, the authors showed that by downregulating Cx43 expression, ET-1 was capable of relieving the inhibition of c-Src, leading to increased HIF1α activity. The rise in HIF1α led to upregulation of GLUT1 and GLUT3 transporters, as well as hexokinase-I and hexokinase-II to promote glucose uptake and utilization through glycolysis and is supported by a recent study (discussed above) demonstrating decreased HIF1α following treatment with TAT-Cx43_266-283_ [179]. These results were later expanded to a connection between Cx43 and angiogenesis. HIF1α induces new blood vessel formation, and factors that promote this process such as members of the vascular endothelial growth factor (VEGF) family, are under the control of HIF1α which would be increased during times of tissue hypoxia. Using the mouse B16f10 melanoma and 4T1 mammary cancer cell lines, expression of Cx43 reduced production of VEGF, while knock-down increased levels, potentially through the connexin/HIF1α/c-SRC axis [121]. Recently, a study examining hypoxia-induced pulmonary hypertension demonstrated HIF1α dependent upregulation of Cx43 [274]. Using both a rat model of hypoxia and cobalt chloride induced hypoxia in pulmonary artery smooth muscle cells (PASMCs), reduced oxygen led to increased expression and phosphorylation of Cx43 at serine 368. This change led to increased proliferation of PASMCs that was blocked by a Cx43 inhibitor peptide, Cx43 knock-down, or use of a HIF1α inhibitor. Furthermore, HIF1α co-immunoprecipitated with the Cx43 promoter, suggesting a transcriptional regulatory network. Based on these divergent studies, it is apparent that context-specific relationships exist between Cx43 and HIF1α. Expanding upon this in the context of cancer will be important to better place the role of connexins in hypoxia induced signaling.

### 5.5. Transcriptional Regulation

An area that will surely provide significant insight to connexins and energy metabolism, is exploration of the numerous transcriptional programs controlled by glucose. Studies such as those by Chen et al. discussed above, are important for connecting connexin gene regulation to substrate metabolism [214]. Additional gene regulators such as the glucose-responsive transcription factors ChREBP (MLXIPL) and MondoA (MLXIP), and the non-histone DNA binding protein HMGA1, contribute to gene regulation and glucose homeostasis, and are largely uncharacterized in the context of direct or indirect connexin gene regulation. However, important relationships in this area are beginning to emerge. Thioredoxin-interacting protein (TXNIP) was originally characterized for its role in cellular redox homeostasis through its interactions with thioredoxin (TXN) [275]. More recently, TXNIP has been shown to play a major role in cellular metabolism with implications on cancer through its inhibition of glycolysis and glucose uptake by downregulating the expression of GLUT1 [276,277,278,279,280,281,282,283,284]. TXNIP acts as a glycolytic sensor and its activation promotes aerobic metabolic pathways and mitochondrial activity, while its inhibition promotes anaerobic glucose utilization. TXNIP exerts these effects, in part, through inhibition of HIF1α and is inversely correlated with PI3K/AKT signaling, while AMPK activity decreases its function [285]. Inhibition of GJIC or siRNA mediated knock-down of Cx43 reduced TXNIP activity and increased GLUT1 expression through a mechanism that involved activation of ERK signaling [286,287]. These results suggest a potential ability of Cx43 to reduce glycolytic metabolism through a TXNIP mediated mechanism, however, little is known about the details of this relationship and data in cancer cells is needed.

Of the numerous specific transcription factors that regulate connexin expression, many are influenced by nutrient accessibility. For example, Iroquois homeobox gene 3 (IRX3) has divergent roles on connexin expression, suppressing Cx43 while upregulating Cx40 in cardiac cells [288]. More recently, IRX3 was found to be downregulated by high glucose levels, indicating a possible transcriptional response for connexin expression mediated by this transcription factor in response to glucose concentrations [289]. Furthermore, important is the fact that many of the basal transcription factors reported to mediate connexin expression can be affected by metabolism of glucose. Studies have shown changes to regulation of the activator protein 1 (AP1) heterodimeric transcription complex in response to glucose in a variety of cell types [290,291,292]. Similar regulation affects transcription factor sp1 (SP1), which has been described as a sensor for glucose levels and is subject to O-GlcNAcylation [203,293,294,295,296,297,298]. Since both of these factors regulate basal connexin expression, it is easy to appreciate the potential for glucose metabolism to alter connexin gene expression. Studies that continue to evaluate these relationships in cancer will provide a more detailed understanding of the metabolic influence on connexin transcriptional responses, and likely uncover a highly integrated network.

Adding to the regulation of connexins at the mRNA level, a growing number of miRNAs have been reported to affect glucose metabolism and an expanding list of connexin transcripts are regulated by miRNAs [299,300,301,302]. For example, miRNA-1 and miRNA-206 control translation of connexin genes and have been implicated in glucose-mediated cellular responses with affects on glycolysis enzymes [303,304,305,306,307,308,309,310,311]. Thus, posttranscriptional regulation adds to the complexity of connexin control mechanisms that are possible through changes in glucose availability.

### 5.6. Extracellular Vesicles and Tunneling Nanotubes

Perhaps one of the most interesting growing areas linking cancer metabolism and connexin regulation involves the role of short-range communication mechanisms. Extracellular vesicles are lipid-bilayer bound particles released from cells and include exosomes which range in size from 30 to 200 nm and originate from endocytic machinery, and microvesicles, which are larger (approximately 100–1000 nm) and result from budding of the plasma membrane [312]. These extracellular vesicles mediate the transfer of a wide range of cargo including lipids, nucleic acids and miRNAs between cells and involve complex mechanisms regarding biogenesis, release and uptake. Their presence influences the local microenvironment and affects cellular signaling, and consequently, cellular behavior. The effects of extracellular vesicles on cancer cell function have been widely documented and this form of signaling has been shown to originate from both cancer cells and stromal cells within the microenvironment [313,314,315]. Seminal work in this area demonstrated that Cx43 was not only present within exosomes, but formed channels that facilitated the transfer of cargo from exosomes into the recipient cells, as well as internalization of the exosomes themselves [316]. Since this report, roles for connexins within extracellular vesicles is continuing to expand [317,318,319,320,321,322]. Regarding cellular bioenergetics, exosomes are capable of altering cancer cell metabolism through the delivery of metabolic pathway intermediates. Strikingly, uptake of CAF derived exosomes by prostate cancer cells decreased mitochondrial metabolism and oxygen consumption rates, while simultaneously inducing glycolysis activity as measured by extracellular acidification and lactate production [323]. The shift from oxidative to glycolytic metabolism also repurposed glutamine for reductive carboxylation within the citric acid cycle. This promoted citrate production leading to palmitate synthesis and subsequent lipid biogenesis while reducing contribution of carbon entry into the citric acid cycle from glucose. Interestingly, these changes did not stem from a shift in oxygen availability and ultimately promoted the Warburg effect. Considering these relationships, further examination of connexins within extracellular vesicles to modulate cancer cell metabolism will add to the growing roles of this dynamic protein family.

**Figure 4:**
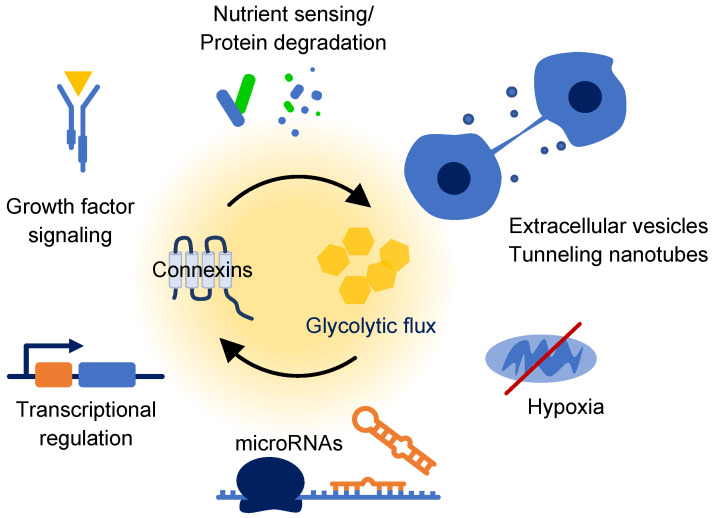
Emerging connections between connexin regulation and glucose metabolism. Data for some relationships is limited in cancer and requires continued research to better define the significance of these associations, which are likely to exhibit interdependence.

Another area receiving attention in the field of cancer metabolism is the role of tunneling nanotubes (TNTs). These thin projections of the plasma membrane are capable of connecting cells over long distances to allow for direct exchange of molecules [317,324,325]. Like extracellular vesicles, a wide array of metabolites are mediated through this type of communication, including the transfer of cellular organelles. At the connecting points of TNTs, numerous mechanisms for the exchange of cellular components have been described including open ended connections between cytoplasm. In close-ended TNTs, gap junctions mediate their normal roles of maintaining electrical coupling and exchange of small molecules and nutrients such as glucose over long distances. These qualities have significant implications on the dynamics of heterogenous tumor architecture and provide a means for direct communication through associated stroma and matrix. Activity of gap junctions during this process keeps the door open to metabolic regulation. Highlighting this point, hypoxic conditions stimulated TNTs in ovarian cancer cells and TNT formation was higher in chemoresistant cells [326]. Additionally, the role of connexins appears to be greater than simply providing a mechanism for communication at the ends of TNTs as connexin proteins may also be necessary for their formation. For example, loss of Cx43 expression reduced the length and number of TNTs in breast cancer cell lines [327]. While much remains unknown regarding the biogenesis and regulation of TNTs, particularly in cancer cells, connexin proteins have already been demonstrated as both functional and regulatory components. This adds to a growing list of glucose-linked metabolic control that needs delineation regarding connexin function (Figure 4).

## 6. Conclusions

Because glucose and the pathways that control its utilization are central to cellular metabolism and interconnected to a multitude of signaling pathways, it is not difficult to find connections between connexin function and glucose metabolism. However, clearly defining the roles, mechanisms and dependencies of this relationship remains a formidable task of cell biology and biochemical analysis, especially within the context of cancer. The constantly shifting metabolism of tumor cells and their fluctuating microenvironment coupled with genetic changes that alter nutrient consumption, present challenges when attempting to recapitulate this indeterminate setting experimentally.

Standard cell culture conditions typically utilize supraphysiological concentrations of nutrients, oxygen pressures and an environment of static versus perfused substrate metabolism without stromal influence or tissue structure. Nonetheless, studying connexin dynamics in a metabolically sequential, albeit controlled environment remains important, while utilization of more advanced culture techniques and metabolomics approaches will be imperative to fine tune what we know about these developing relationships [234,328,329]. Furthermore, incorporating this knowledge into the scope of other metabolic substrates remains vital as any one metabolite cannot be studied in isolation and each metabolic shift creates ripple effects that alter molecular regulation. Additionally, while a large volume of the data presented here focuses on Cx43, continued expansion on the role of other connexins in cancer metabolism is necessary and will lead to important insight.

It is clear that connexins play multiple roles in controlling glucose (and cellular) metabolism while responding to environmental and intracellular nutrient sensing. More roles will be described as attention to this area continues to increase. Importantly, the growth of knowledge regarding these associations will shed light on clinical and therapeutic implications.
